# Orthodontic treatment of an unerupted mandibular canine tooth in a patient with mixed dentition: a case report

**DOI:** 10.1186/s13256-016-0923-6

**Published:** 2016-06-10

**Authors:** Maria Teresa Dinoi, Enrico Marchetti, Umberto Garagiola, Silvia Caruso, Stefano Mummolo, Giuseppe Marzo

**Affiliations:** 1 Department MeSVA, School of Dentistry, University of L’Aquila, L’Aquila, Italy; 2 Biomedical Surgical and Dental Sciences Department, University of Milan, Milan, Italy

**Keywords:** Unerupted, Orthodontics, Canine tooth, Mixed dentition

## Abstract

**Background:**

The aim of this case report was to describe the surgical–orthodontic treatment of an unerupted mandibular canine tooth in a 9-year-old girl.

**Case presentation:**

A 9-year-old white girl presented with an unerupted right mandibular canine tooth. Combined surgical–orthodontic treatment was performed to correct dental impaction and to achieve good aesthetic and functional results.

**Conclusion:**

Orthodontic treatment achieved all of the required objectives.

## Background

Dental eruption is a dynamic and complex biological and physiological process that occurs over several years. The process includes the formation of teeth and their migration in the jaws until their eruption in their final functional position in the dental arches. The age at which the temporary and permanent teeth appear varies markedly among individuals and can be related to several factors, including gender, dentition, socioeconomic status, and height.

Under certain anatomical conditions, trauma or infective processes involving the deciduous teeth can cause alterations of their eruptive process, preventing the permanent tooth from appearing in the oral cavity within the physiological eruption timeframe or causing ectopic positioning. A tooth is considered “impacted” when it fails to erupt in the dental arch within the expected developmental window. Teeth may become impacted because of adjacent teeth, dense overlying bone, excessive soft tissue, or genetic abnormalities. The reported incidence of dental impaction varies between 5.6 and 18.8 %, with a higher frequency among women [1]. The third molars are most frequently impacted (20 to 30 %) because they are the last teeth to erupt in the oral cavity, followed by the maxillary canines (85 % with palatal dislocation), mandibular second premolars (0.3 %), and central maxillary incisors (0.1 %) [2–5].

Several classifications can be used to evaluate the degree of tooth impaction. These classifications are based on different factors, such as duration of impaction (that is, temporary versus permanent), number of impacted teeth (that is, single versus multiple) [6], the degree of impaction (that is, total versus partial) [6], and cause of impaction (that is, primary versus secondary). Primary impaction is caused by intrinsic factors, such as tooth anatomy and tilt, whereas secondary impaction is caused by external factors, such as cystic lesions, supernumerary teeth, and neoplasms [6].

The etiopathogenesis of dental impaction is vast. Causes of dental impaction can be classified as general, local, structural, and systemic. General causes include genetics, endocrine hypofunction or hyperfunction, metabolic dysfunction, and infectious diseases [7]. Local causes include obstructed eruption, lack of space, ankylosis of primary or permanent teeth, ectopic position of the tooth bud, dilacerations of the roots, soft tissue or bony lesions, fibrosis, and habits [7]. Structural causes include maxillary hypoplasia, severe hyperdivergence, skeletal open bite, and congenital pathologies of the maxillofacial system [7]. Systemic factors include prenatal causes (heredity), postnatal tuberculosis, anemia, malnutrition, and endocrine disorders of the thyroid or parathyroid gland.

Several therapies are possible for impacted teeth, including classic orthodontic treatment, combined surgical–orthodontic treatment, conservative surgery, and radical surgical treatment [7]. In the simplest cases of tooth retention, conventional orthodontic treatment should be chosen. When the impacted tooth has anomalies of location and inclination or a particular coronoradicular morphology, combined surgical–orthodontic treatment should be chosen. When tooth eruption is hampered by a pathological condition, such as a cyst or odontoma, and the tooth’s position in the arch depends on removal of the obstacle, conservative surgical treatment should be selected. In the case of serious anomalies in tooth anatomy or location, or at the patient’s request, radical surgical treatment (extraction) may be chosen. Maintaining the teeth in the arch is important, to ensure that the patient will have adequate functionality and good aesthetics.

## Case presentation

**Fig. 1:**
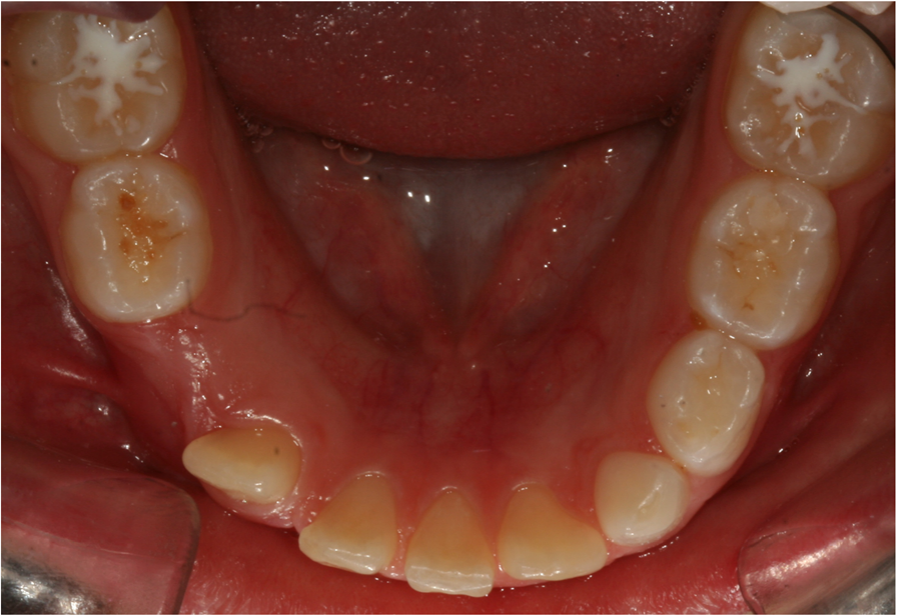
Initial intraoral photograph of the mandible

This case report describes the case of a 9-year-old white girl with mixed dentition. An extraoral examination revealed no significant facial asymmetry. An intraoral examination showed dentition appropriate for her age (Fig. 1). She had no family or medical history that would explain eruption abnormalities.

**Fig. 2:**
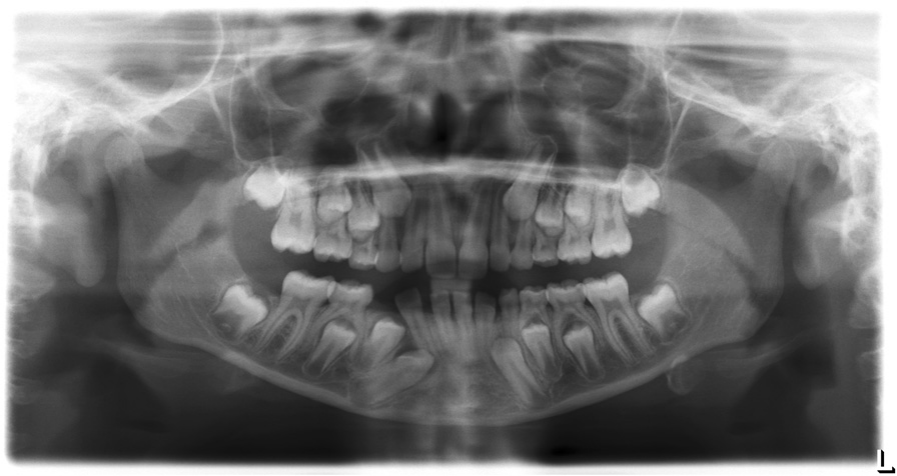
Initial panoramic radiograph

Orthopantomography of her dental arches and a lateral teleradiograph of her cranium were performed for cephalometric evaluation to allow planning of an appropriate treatment plan. Orthopantomography showed an unerupted right canine tooth. As shown in Fig. 2, the canine tooth still had eruptive capacity but no physiological eruptive path was present.

At her age, functional treatment is generally advisable. However, considering the canine ectopia, surgical–orthodontic treatment was chosen to move the canine into her arch. This case shows that it is important to act early, during the mixed dentition phase, to prevent worsening impaction of ectopic teeth, which could require tooth extraction at a later stage. The treatment involved creating a surgical incision next to her unerupted canine, applying traction on the tooth toward her arch with an anchoring device and bonding of her lower arch, followed by a phase of functional orthodontics to improve the shape of her arches.

**Fig. 3:**
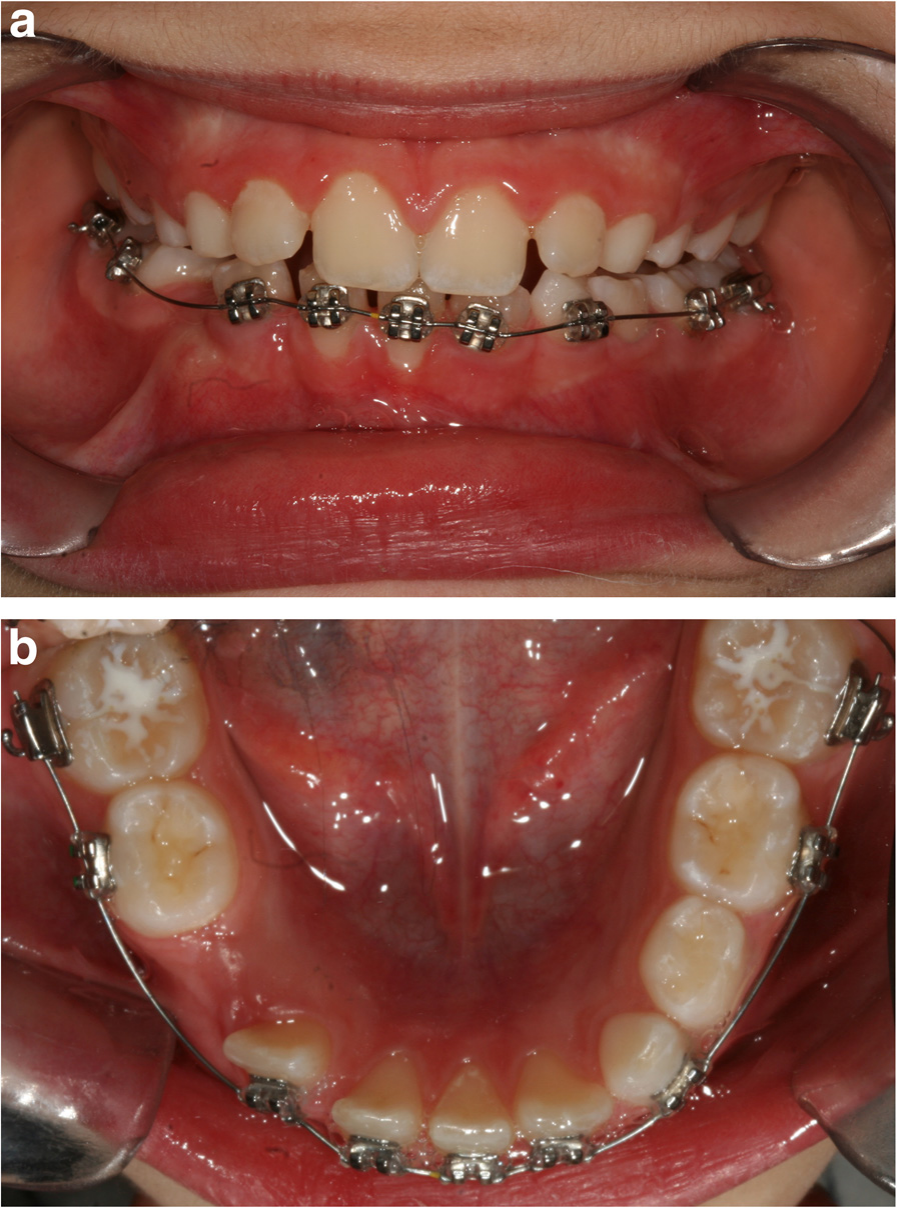
**a b** Banding of the lower arch

The first session involved bonding her lower arch with prepressed and pre-angled attachments to align the four incisors. The first archwire used was 0.014-inches (0.356 mm) round archwire made of nickel and titanium. Bonding was performed using her primary teeth to provide a greater anchor (Fig. 3a, b).

**Fig. 4:**
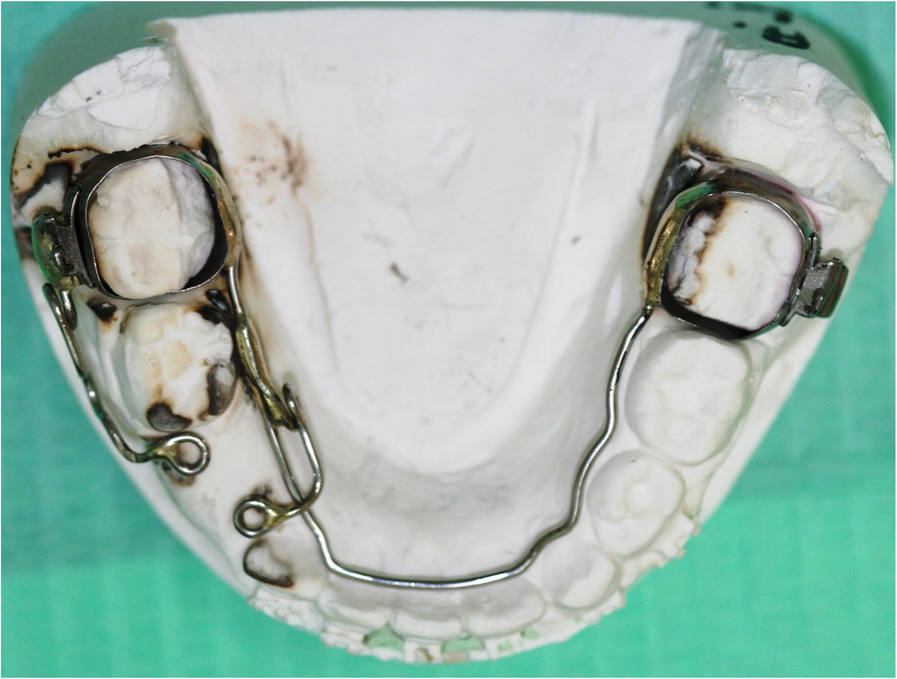
Lingual arch

In the next session, we replaced the round archwire with 0.016×0.022-inches (0.406×0.559 mm) rectangular archwire made of nickel and titanium. A dental impression was made with the orthodontic bands on her mandibular sixth tooth to build a mandibular lingual archwire than eyelet in area 43, which was necessary to apply traction to her impacted tooth (Fig. 4).

**Fig. 5:**
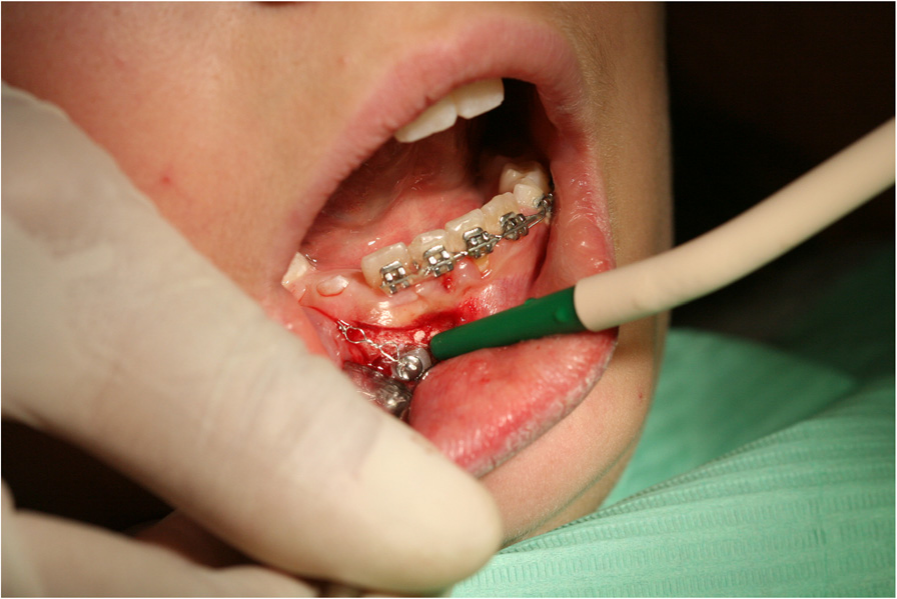
Surgical opening

Twenty days later a surgical opening was made (Fig. 5). A button was placed at the coronal level of her unerupted tooth and was tied with elastic thread to the eyelet of the auxiliary appliance to provide traction. The lingual archwire was cemented after the surgical opening.

**Fig. 6:**
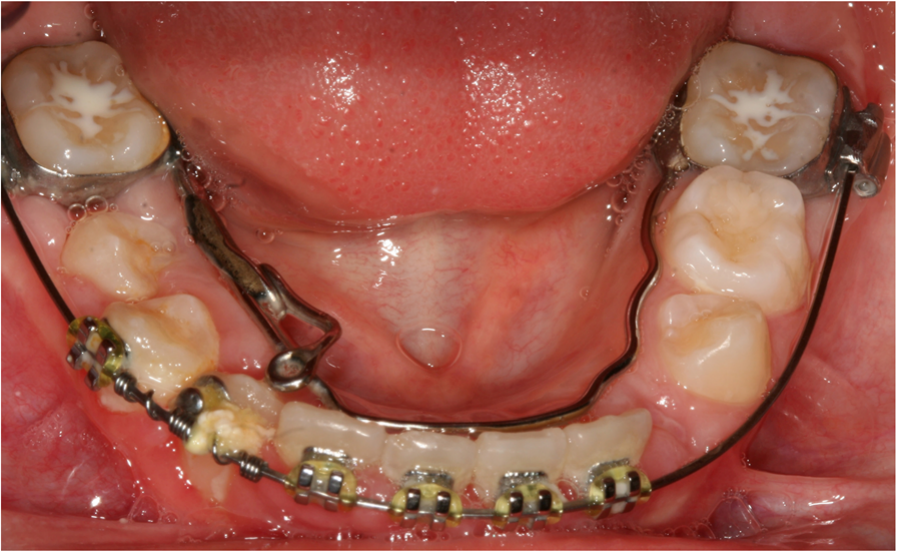
Canine tooth visible in the arch

Traction was applied slowly, with replacement of the elastic thread every 15 days. Approximately 4 months after the surgical opening was made, the tooth became visible in her arch (Fig. 6). Traction on the tooth continued to guide it to its physiological seat. The button was replaced with a prepressed and pre-angled attachment.

**Fig. 7:**
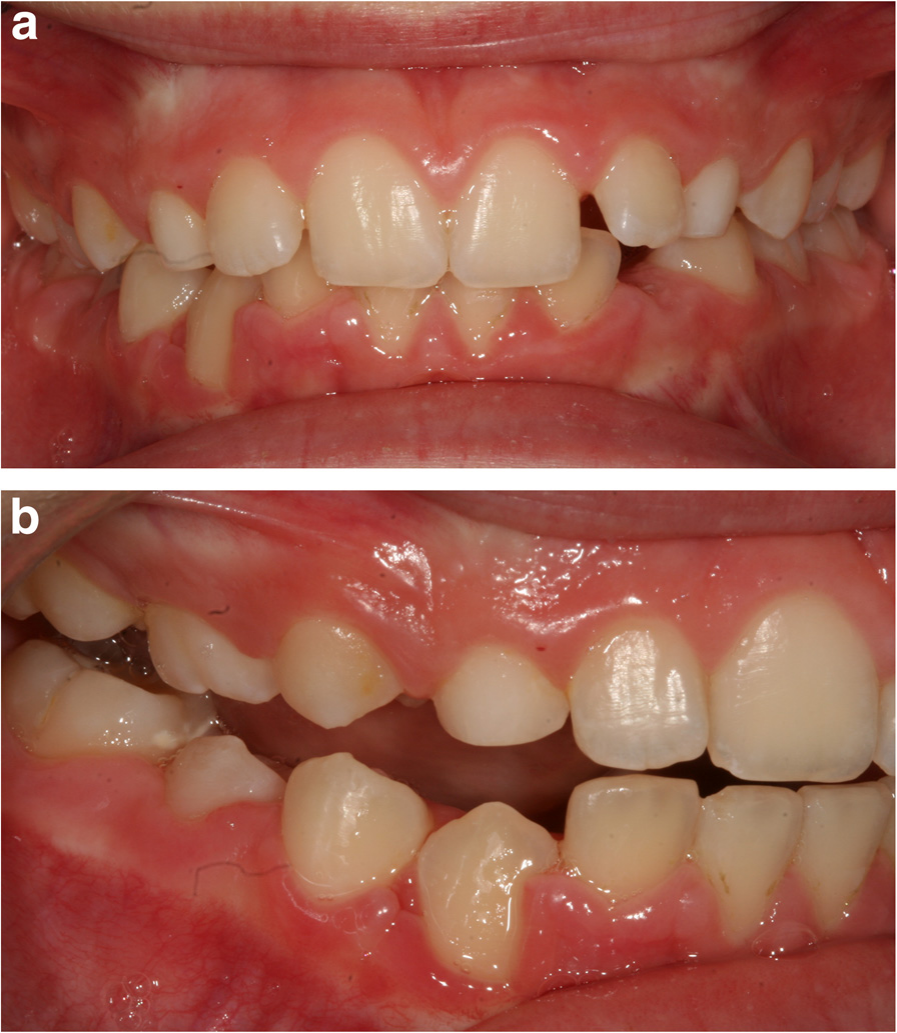
**a** Front final photographs, **b** Lateral final photograph

Approximately 8 months after surgery, the tooth had moved to its physiological location and the bands were removed from her lower arch (Fig. 7a and b).

**Fig. 8:**
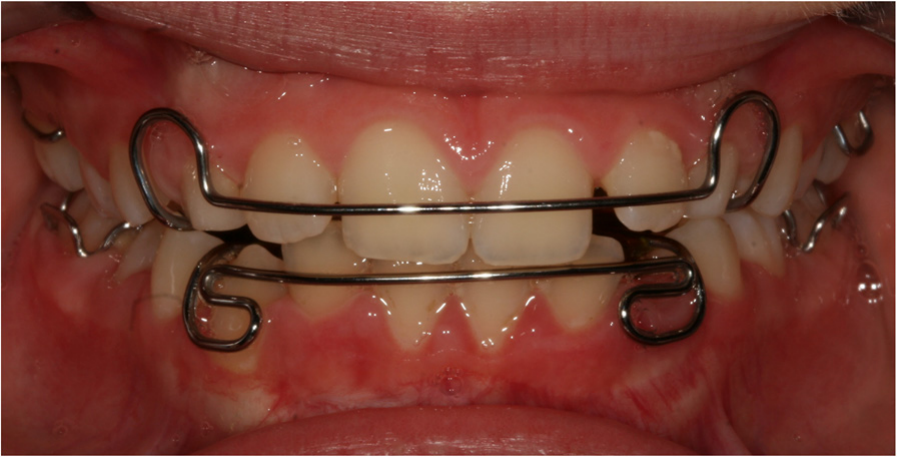
Schwarz retainers

Orthodontic treatment (Fig. 8) was continued with two Schwarz appliances to slowly expand her arches and to improve their shape, postponing the final alignment of her teeth to a later stage, when her dentition will be complete.

## Conclusions

The purpose of this case report was to describe the combined surgical–orthodontic treatment of an unerupted mandibular canine tooth in a 9-year-old girl. Given her age, functional treatment would generally be advisable to expand her arches. In this case we preferred to immediately implement fixed treatment to bring the canine into her arch and to avoid the risk of the tooth requiring extraction with delayed treatment. The treatment was successful, with recovery of the impacted canine. Good aesthetic and functional results were achieved.
